# Supplementary material for: Conserved and Noncanonical Activities of Two Histone H3K36 Methyltransferases Required for Insect-Pathogenic Lifestyle of Beauveria bassiana
Source: J Fungi (Basel). 2021 Nov 11;7(11):956. doi: 10.3390/jof7110956 (PMC8623635; doi:10.3390/jof7110956)
Supplement: Supplementary file 1 [file jof-07-00956-s001.zip › jof-1441567-supplementary.pdf]

Supplementary Material

Conserved and Noncanonical Activities of Two Histone H3K36 Methyltransferases Required for Insect-Pathogenic Lifestyle of *Beauveria bassiana*

Kang Ren, Ya-Ni Mou, Sheng-Hua Ying and Ming-Guang Feng *

MOE Laboratory of Biosystems Homeostasis & Protection, College of Life Sciences, Zhejiang University, Hangzhou 310058, China; 11807120@zju.edu.cn (K.R.); 11907036@zju.edu.cn (Y.-N.M.); yingsh@zju.edu.cn (S.-H.Y.)
* Correspondence: mgfeng@zju.edu.cn

Table S1. Paired primers used for targeted gene manipulation of *set2* and *ash1* in *B. basssiana*.

Primers	Paired sequences (5′–3′) [a]	Purpose [b]
Set2-F/R	CAATCACAAACACCTTCAAAATGAAGGATGACGAGAACGCGACGGTCGAG / CTCGCCCTTGCTCACCATGTGACTCAGAACCTCCTGTTTTCGATTCTG	Cloning *set2* cDNA (2700 bp) for fusion to *gfp*
Ash1-F/R	CAATCACAAACACCTTCAAAATGTTGCCACTCCTTCTCGAACCGCAGTCG / CTCGCCCTTGCTCACCATGTCGTCCACCAATTTTATTGTGGGCTTGG	Cloning *ash1* cDNA (2475 bp) for fusion to *gfp*
upSet2-F/R	ACGAGCTGTACAAGTAACCCGGGGCATCTCAAGCAAAAAAGGG / TGGCTGCAGGTCGACGGATCCCAGAGGAGGCTCGCAAACAC	Cloning 3′ *set2* (1420 bp) for targeted gene disruption
dnSet2-F/R	GACCCATGGCTCGAGTCTAGATTCTGACTGTCTCTATGGCTCC / GGTGGTGGTGGCTAGCGTTAACGTCGCTCTGCTGAATACGGT	Cloning 5′ *set2* (1005 bp) for targeted gene disruption
flSet2-F/R	ATCCGTCGACCTGCAGCCAAGCTTCCCCCATTTCTGTTCTCCAT / ACACTAGTCAGATCTTCTAGTGTTAGCATACACGGGCGTTTTT	Cloning full-length *set2* (6645 bp) for complementation
upAsh1-F/R	ACGAGCTGTACAAGTAACCCGGGAGATGGTCGGCTTGATGAGG / TGGCTGCAGGTCGACGGATCCGCAGAGGCAGGGTAGAATGA	Cloning 3′ *ash1* (1278 bp) for targeted gene disruption
dnAsh1-F/R	GACCCATGGCTCGAGTCTAGACGCTAATGGCGATGATGT / GGTGGTGGTGGCTAGCGTTAACCACGGTCCGATGTCTTGATA	Cloning 5′ *ash1* (1119 bp) for targeted gene disruption
flAsh1-F/R	ATCCGTCGACCTGCAGCCAAGCTTCGGAAGATTGAATAATGTGAAG / ACACTAGTCAGATCTTCTAGTGTCGCCCAGATAGCAAGAAA	Cloning full-length *ash1* (5563 bp) for complementation
pSet2-F/R	CGCATCGTCAATCCCAGTCT / AAATCGTTGGCGGCTAAATC	PCR detecting *set2*
pAsh1-F/R	CTCACACCAAACGCCAACT / CGCCCACACTACCACCAG	PCR detecting *ash1*
qSet2-F/R	GAGATCCCCGTCGACAGAG / CCTCAGAGGTCATATCGGGG	qPCR detecting *set2*
qAsh1-F/R	TTGCCACTCCTTCTCGAACC / GCTCAGATGGCAGTTCAGTG	qPCR detecting *ash1*

[a] Underlined regions denote introduced the cleavage sites of two pairs of restriction enzymes (*Xma*I/*Bam*HI and *Xba*I/*Hpa*I) for targeted gene disruption through homologous recombination of the bar-separated 5′ and 3′ fragments of *set2* or *ash1* and the recognition fragments for gateway exchange to construct the complementation vector.

[b] PCR detection aimed at the fragments of 1508 bp for deleted *set1* and 1049 bp for WT and of 1289 bp for deleted *ash1* and 937 bp for WT.

Table S2. Antibodies used for western blotting of histone H3 and mono-, di- and trimethylated H3 lysines.

Name of antibody product	No. catalog	Manufacturer*	Purpose
Histone H3 (D1H2) XP Rabbit mAb	4499S	CST	Anti-histone H3
Mono-Methyl-Histone H3 (Lys4) (D1A9) XP Rabbit mAb	5326T	CST	Anti-H3K4me1
Di-Methyl-Histone H3 (Lys4) (C64G9) Rabbit mAb	9725T	CST	Anti-H3K4me2
Tri-Methyl-Histone H3 (Lys4) (C42D8) Rabbit mAb	9751S	CST	Anti-H3K4me3
Mono-Methyl-Histone H3 (Lys9) Rabbit Polyclonal	39887	Active Motif	Anti-H3K9me1
Di-Methyl-Histone H3 (Lys9) (D85B4) XP Rabbit mAb	4658T	CST	Anti-H3K9me2
Tri-Methyl-Histone H3 (Lys9) (D4W1U) Rabbit mAb	13969S	CST	Anti-H3K9me3
Anti-Histone H3 (mono methyl K36) Rabbit Polyclonal	ab9048	Abcam	Anti-H3K36me1
Di-Methyl-Histone H3 (Lys36) (C75H12) Rabbit mAb	2901T	CST	Anti-H3K36me2
Tri-Methyl-Histone H3 (Lys36) (D5A7) XP Rabbit mAb	4909S	CST	Anti-H3K36me3

* CST, Cell Signaling Technology (Boston, MA, USA); Active Motif (Shanghai, China); Abcam (Shanghai, China).

Table S3. Paired primers used for transcriptional profiling of phenotype-related genes in *B. bassiana*.

Gene	Tag locus*	Annotation	Sequences (5′–3′) of paired primers
**Used as reference gene**			
GADPH	BBA_05480	Glyceraldehyde-3-phosphate dehydrogenase	TCGAGTCCACTGGTGTCTTC / CCCTCAACAATGCCGAACTT
**Involved in nutritional uptake, conidiation and hydrophobicity**			
cre1	BBA_05136	Carbon catabolite repressor	TCGAGCACCAGACAAGACAC / CTTGTTACCACGGCGAGAGT
brlA	BBA_07544	Developmental activator BrlA	GATGGATGACAAGTGCGATG / AAACTCGCACGAGAAACGAT
abaA	BBA_00300	Developmental activator AbaA	GCAAGTCTCCAGCCATAT / CTCCTCTTCGTCATAGTAGTC
wetA	BBA_06126	Developmental activator WetA	ATGCGGTACTACAGCCAAGG / GAGTTCCTGCTGGCTACTGG
vosA	BBA_01023	Developmental activator VosA	ACTCATGGGCTCATTGGTGG / CCGGCAAGAGAGATCCGAAA
hyd1	BBA_03015	Class I hydrophobin Hyd1	ATGGTGGAAAGGATCTGCAC / TGGGAAAGAAGACCATCAGC
hyd2	BBA_06599	Class II hydrophobin Hyd2	TGTCAAGACTGGCGACATTT / ATTGGGACAAGCTGGTTGAG
hyd3	BBA_00530	Hydrophobin-like protein Hyd3	CTGGCCACCCTACTTCTGTC / TCTGGCTAGGGGTAGAGCAA
hyd4	BBA_03071	Hydrophobin-like protein Hyd4	AGTGCTGTGCCACTGACATC / GGGGTCATGCAAAAGAGACT
hyd5	BBA_02999	Hydrophobin-like protein Hyd5	GAGGCTCGCACTGATAAAGC / CAACCTTGGCACAAATTTCC
**Involved in antioxidant activity**			
cat1	BBA_06186	Spore-specific catalase CatA	CAACAACATCCCCGTCTTCT / ACACCAAATCCCTGCATCAT
cat2	BBA_05603	Secreted catalase CatB	CTCGTACTTGGACACGCAGA / TTGTTGAGGGTGTTTGGTGA
cat3	BBA_09109	Cytoplasmic catalase CatC	CGCACAAGAGAACCTTCACA / AATGGTTTGTGTGTCGCTGA
cat4	BBA_09760	Secreted peroxidase/catalase CatD	TCTCTGTCCTGGGCTGATCT / CTTGCTGGGGACAAACTCAT
cat5	BBA_09338	Peroxisomal catalase CatP	CAAGGATTCTTCTGGCAAGC / AGCAATGAGAGCAACGGTCT
cat6	BBA_06567	Catalase-like protein, heme-dependent	TTTCCGTGAGGACATTCACA / AGGACTTTTCGTCCCCATCT
sod1	BBA_02311	Cytosolic Cu/ZnSOD	GCGGCTTCCACATCCACACCTTTG / GGTCCAGCGTTGCCAGTCTTGAG
sod2	BBA_09706	Cytosolic MnSOD	CCAGTGTTTGGCATTGACATG / TCAGCCGTCTTCCAGTTGATG
sod3	BBA_09382	Mitochondrial MnSOD	TCTCCGGCAAGATTATGGAGC / TTGGCGTCATTCTTGGCCT
sod4	BBA_04317	Mitochondrial FeSOD	CGAGATGGTCCTTACGGCTTCAG / GCTCCCAGGTGTTGAGGCATAG
sod5	BBA_01984	Cell wall-anchored Cu/ZnSOD	CGGCGACCTCAGCGGCAAGTAC / GCCAGCAACAACAGGGACCGTAGG
**Involved in cell wall composition and signaling**			
bck1	BBA_01318	MAP kinase kinase kinase Bck1	TAGGGACTGGATTTGGCATC / CTGCAGCAGAACTACGCTTG
mkk1	BBA_01095	MAP kinase kinase 1 Mkk1	GGTGAAGCCAACACCTTCAT / GCAAGTCAATCAGACCAGCA
slt2	BBA_03334	MAP kinase Slt2	TCGACAAGATGCTGGCCTTT / ATGTGCAGGTAAGGGTGCTC
smi1	BBA_06704	SMI1/KNR4 family protein	ACCGACATTCCTCGTTTGAC / AAGAGCTGTTGGGAGTGGAA
fks1	BBA_10207	Beta-1,3-glucan synthase catalytic subunit	GAGCCAGTCGAGGAGTTCAC / TCGGGGTACCAGAAAAGTTG
chs1	BBA_03793	Chitin synthase chaperone-like protein Chs7	GAATCGAGCCCTCATGCTAC / TCAACAACGAGCGAGAAGAA
chs2	BBA_02360	BRCA1 C Terminus domain-containing protein	TAGACGCCTGCGAGAAGAAT / AATCACGCTCTCATCCTCGT
chs3	BBA_07346	Class VII chitin synthase	GCTCTGTCAAGGTGCTTTCC / GAGGTTGTGGGTGACGAGTT
chs4	BBA_06845	MIF4G domain-containing protein	GATGGCAATCGATTTTTGCT / TCAACAAGACCCTTGGGAAC
chs5	BBA_06859	Class V chitin synthase	CTGGTGAGAAGGGCAAGAAG / GCATAGCTGTAGCCCGAGAC
chs6	BBA_04667	Chitin synthase 3a	AGGGCTCTGTCCTTTCCATT / CGCAAATCGTAACCGTTTTT
Chs7	BBA_03590	Chitin synthase 1	GCCAAAGACTTGCTCGGTAG / GGCGGCGAACATAAGATAAA
chs8	BBA_08396	Glycosyltransferase family 2	TTCAAGAACTCTGCCCACCT / CAGCGGAGCTAAAGGAAATG
chs9	BBA_03236	Class 2 chitin synthase	TCCCTTGCCAGATGATTTTC / ATCAAAGGCCTTCCAAAGGT
**Involved in HOG signal pathway regulating osmotic response**			
sskB	BBA_00937	MAP kinase kinase kinase SskB	GCTCACCACATTTCAGCGAA / TGCAGACCGTCTTCCTTCAT
pbs2	BBA_02330	MAP kinase kinase Pbs2	GCAGAAAAGGGGTGGATCAC / GTGTGCCGAACCTTGTAGAC
hog1	BBA_05209	MAP kinase Hog1	CCTTCACAGACTCCTCACGT / GTGTGCCGAACCTTGTAGAC

* Gene accession codes of *B. bassiana* genome under the NCBI accession NL_ADAH00000000.

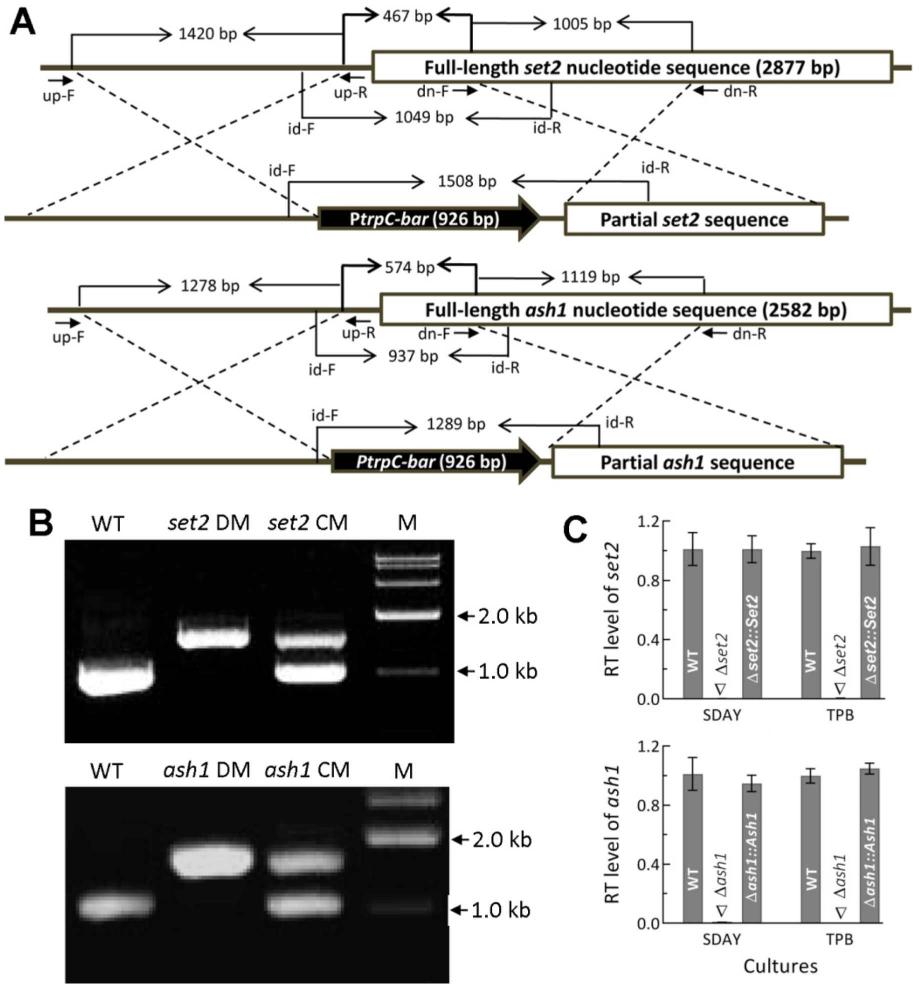

Figure S1. Generation and identification of *set2* and *ash1* mutants in *B. bassiana*. (**A**) Schematic diagrams for the disruption strategy of *set2* and *ash1*. (**B**) The *set2* and *ash1* mutants identified through PCR analysis with paired primers (Table S1). WT, wild-type DNA. DM, DNA of disruption mutant; CM, DNA of complementation mutant. M, molecular ladder of DNA. Detected PCR bands are the fragments of 1508 bp for deleted *set2* and 1049 bp for WT, of 1289 bp for deleted *ash1* and 937 bp for WT, and of both for CM, leading to the disruption of *set2* or *ash1* by deleting a partial promoter/coding fragment of 467 bp (1049 + 926 – 1508 = 467 bp) or 574 bp (937 + 926 – 1289 = 574 bp) as expected in the diagrams. (**C**) Relative transcript (RT) levels of *set2* and *ash1* in the 3-day-old SDAY and TPB cultures of the mutants with respect to the WT standard. Note that the expression of each target gene was completely abolished in DM and well restored in CM. Error bars: standard deviations of the means for three cDNA samples derived from the independent SDAY or TPB cultures and analyzed via qPCR with paired primers (Table S1).

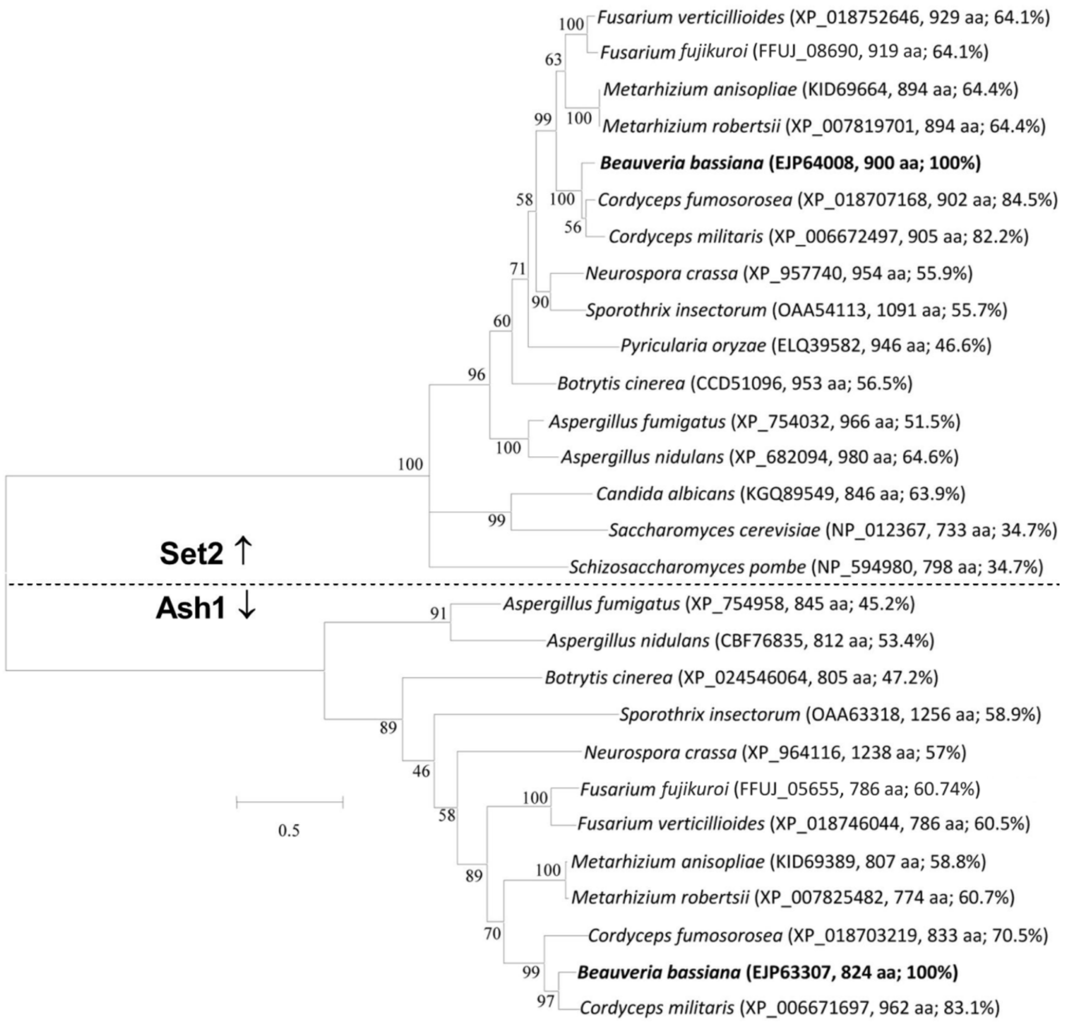

Figure S2. Phylogenetic analysis of ascomycetous Set2 and Ash1 homologs. The tree is constructed with the maximum likelihood method in MEGA7 at http://www.megasoftware.net/. Bootstrap values of 1000 replications are shown at nodes. Scale: branch length proportional to genetic distance. For each fungus, the NCBI accession code of each homolog, the length of its amino acid sequence, and its protein sequence identity to the homolog of *B. bassiana* are given in the parentheses following the fungal nane.
